# Riverbed erosion of the final 565 kilometers of the Yangtze River (Changjiang) following construction of the Three Gorges Dam

**DOI:** 10.1038/s41598-018-30441-6

**Published:** 2018-08-09

**Authors:** Shuwei Zheng, Y. Jun Xu, Heqin Cheng, Bo Wang, Wei Xu, Shuaihu Wu

**Affiliations:** 1 State Key Lab of Estuarine & Coastal Research, East China Normal University, Shanghai, 200062 China; 2 School of Renewable Natural Resources, Louisiana State University Agricultural Center, 227 Highland Road, Baton Rouge, LA 70803 USA

## Abstract

The world’s largest hydropower dam, the Three Gorges Dam (TGD), spans the upper Yangtze River in China, creating a 660-km long and 1.1-km wide reservoir upstream. Several recent studies reported a considerable decline in sediment load of the Lowermost Yangtze River (LmYR) and a rapid erosion in the subaqueous delta of the river mouth after the closure of the TGD in 2003. However, it is unknown if the TGD construction has also affected river channel and bed formation of the LmYR. In this study, we compared bathymetric data of the last 565 kilometers of the Yangtze River’s channel between 1998 and 2013. We found severe channel erosion following the TGD closure, with local riverbed erosion up to 10 m deep. The total volume of net erosion from the 565-km channel amounted to 1.85 billion m^3^, an equivalent of 2.59 billion metric tons of sediment, assuming a bulk density of 1.4 t/m^3^ for the riverbed material. The largest erosion occurred in a 100-km reach close to the Yangtze River mouth, contributing up to 73% of the total net eroded channel volume.

## Introduction

Riverine sediment reduction due to human activities is a phenomenon observed in many river systems around the world in the past several decades^1–4^. For instance, Yang and others^5^ reported a 100 megatons (MT)/year decline in sediment load of the Yangtze River from the 1950 s to the 1990 s; Meade and Moody^6^ found a 3.5 times decrease in sediment load of the Mississippi River from the early to the late 1900 s. The coastal land loss of many river deltas, especially large river deltas such as the Nile^7^, Yangtze^5^, Mississippi^6^, Yellow^8^, and Mekong^9^ deltas, has been attributed to sediment reduction, together with subsidence and sea level rise. These large river deltas are densely populated and are transportation hubs and industrial and commercial centers. Continuous land loss of the deltas is a risk to the sustainability of national economy and global commerce.

Riverine sediment reduction and sea level rise may also affect channel and riverbed dynamics of alluvial rivers in their tide-affected reaches^8,10^. Excessive changes in channel formation possess a threat to the long-term stability of the river delta and its continental shelf. A recent study found that the subaqueous delta of the Yangtze River is undergoing severe erosion owing to the drastic decline of sediment discharge^11^. The Yangtze River used to deliver 500 Mt sediment annually (before the 1970 s) to its 20,000 km^2^ delta plain system^12,13^. Dam construction in the river basin is one of the main reasons that have led to sediment reduction^3,14,15^. In the past half-century, more than 50,000 dams and reservoirs were built in the river’s headwaters and tributaries^16^. During the period between the 1980 s and 1990 s, the world’s largest hydropower dam, the Three Gorges Dam (TGD), was constructed in the upper Yangtze River. As a result, riverbed deformation has been observed in some short reaches downstream of the TGD in recent years. Studies have reported various significant downstream changes following the closure of the TGD, such as sediment reduction^17^, channel adjustment^18,19^, delta recession^11^, flood pulse reduction^12^, and decline of riverine wetland and lake areas^20^. Although a recent study showed that the new long-term hydro-morphological equilibrium may have been established in the middle and lower Yangtze^21^, it is largely unknown how the sediment reduction may have affected channel morphology and sediment transport throughout the Lowermost Yangtze River, which flows through China’s most densely populated and most industrialized region before entering the East China Sea.

The Lowermost Yangtze River (LmYR), starting at Datong (river kilometer, or RK 565) and ending at the river mouth Wusongkou (RK 0), is a tide-affected river reach. Datong has the most seaward gauging station of the Yangtze River with long-term hydrological records, and both water and sediment discharges at this station have been considered as the final input into the East China Sea^22,23^. A few recent studies reported different erosion and deposition patterns near the river mouth, attributing the changes to the reduction in riverine sediment following the Three Gorges Dam closure in 2003^24^. It is important to understand how sediment decline may have influenced the entire channel morphology of the tide-affected river reach from the river’s mouth to Datong. Such knowledge is crucial for predicting long-term stability of the large alluvial river and its deltaic and nearshore continental shelf development.

With the above in mind, this study utilized two sets of channel bathymetric charts of the Lowermost Yangtze River from Datong to Wusongkou, produced 5 years before and 10 years after the TGD closure, to investigate changes in channel morphology and riverbed deformation. Our primary goal was to quantify spatially-explicit erosion and deposition rates along this 565-km long alluvial river reach, in order to elucidate the possible future trend of the TGD effects on the Lowermost Yangtze River.

## Study Area

**Figure 1:**
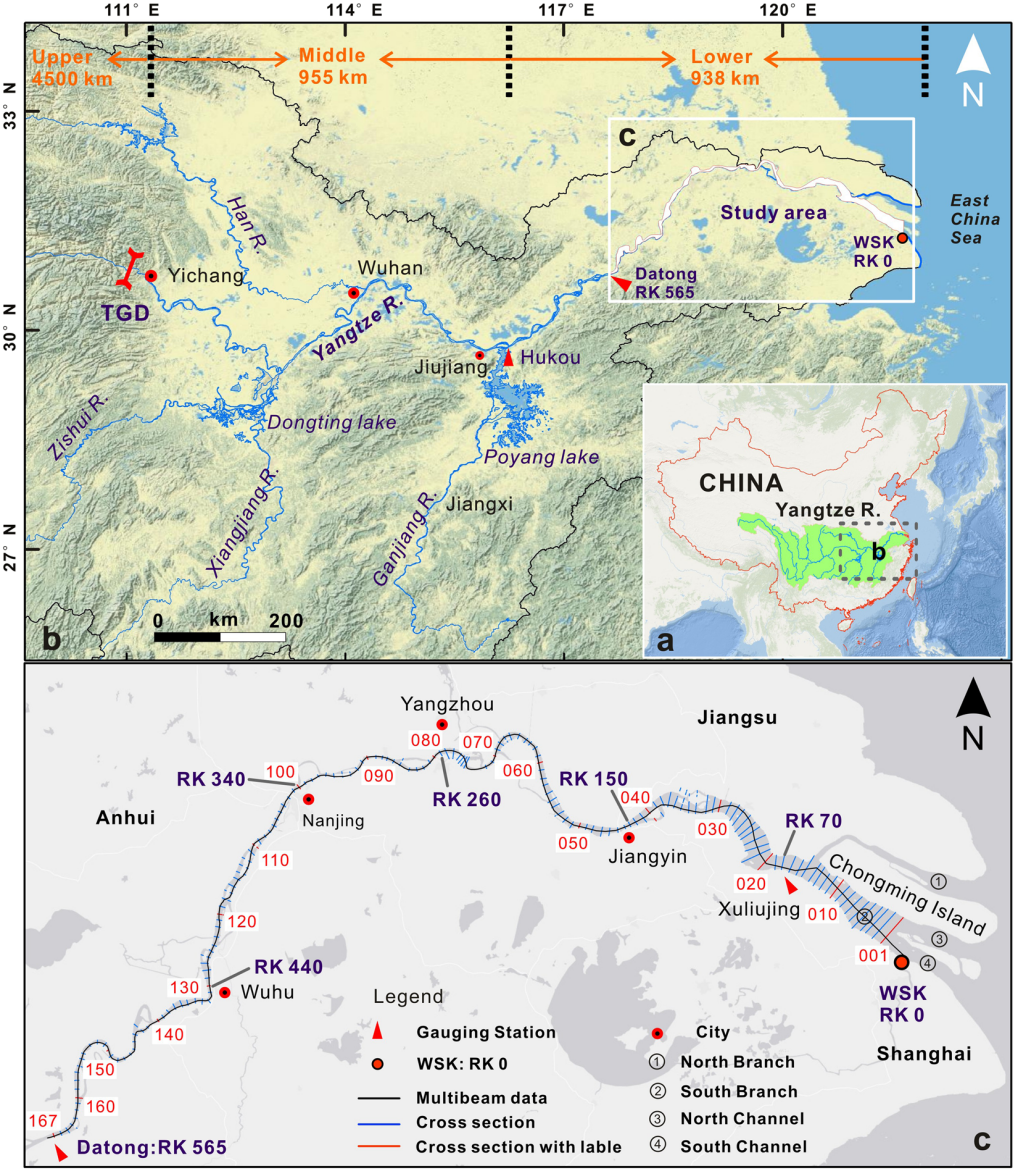
Maps of the study reach of the Lowermost Yangtze River. (**a**) The Yangtze River Basin in China. (**b**) The Middle and Lower Yangtze River. (**c**) The final 565 kilometers of the YR from Datong to Wusongkou with 167 cross sections and the multi-beam bathymetry data collected along the main navigation channel. These cross-sectional measurements were used to assess changes in river channel width and depth between 1998 and 2013. RK: river kilometer. Wusongkou: the 0-km point of the YR, i.e., RK 0. Note: the maps were created using ArcGIS 10.4 (www.esri.com) and CorelDRAW Graphics Suite 2017 (www.coreldraw.com).

The Yangtze River originates in Tibet and enters the East China Sea with a total length of 6300 km. Its upstream, midstream, and downstream reaches extend, respectively, from the headwater to Yichang, from Yichang to Hukou, and from Hukou to the river mouth (Fig. 1). This study investigated the final 565-km river reach of the Yangtze River from Datong to Wusongkou near the river’s mouth to the East China Sea (Fig. 1). The Ghiangnania and Huaiyang shields controlled the main geological structure and a series of fracture zones exist in the study area. Thus, the river morphology and movement trend were controlled by these geological structures^25^. In addition to carrying the flow from the upper Yangtze River basin, this river reach also drains approximately 6% of the entire 1.8 million km^2^ Yangtze River Basin area with a mean precipitation of approximately 1200 mm per year^13^. This region is one of the most densely populated and industrialized areas in China, contributing 24% of China’s national GDP.

Datong is the most seaward gauging station, and discharge at the location is generally considered as the final freshwater flow into the East China Sea (Fig. 1). The annual average discharge at Datong for the period of 1950–2002 is 905 × 10^9^ m^3^ (or 905 km^3^), with an average annual sediment load of 0.38 × 10^9^ tons (or 368 million tons) (www.cjh.com.cn/pages/nsgb). Due to dam construction and soil conservation in the upper Yangtze River Basin, the annual sediment load has declined sharply^11^, particularly after the closure of the Three Gorges Dam (TGD) in 2003. The average annual sediment load during 2003–2015 was 0.14 × 10^9^ tons which is only about one-third of the long-term average sediment load before 2003, while the average annual river flow during 2003–2015 remained similar to the previous long-term average river flow.

## Materials and Methods

### Datasets

**Figure 2:**
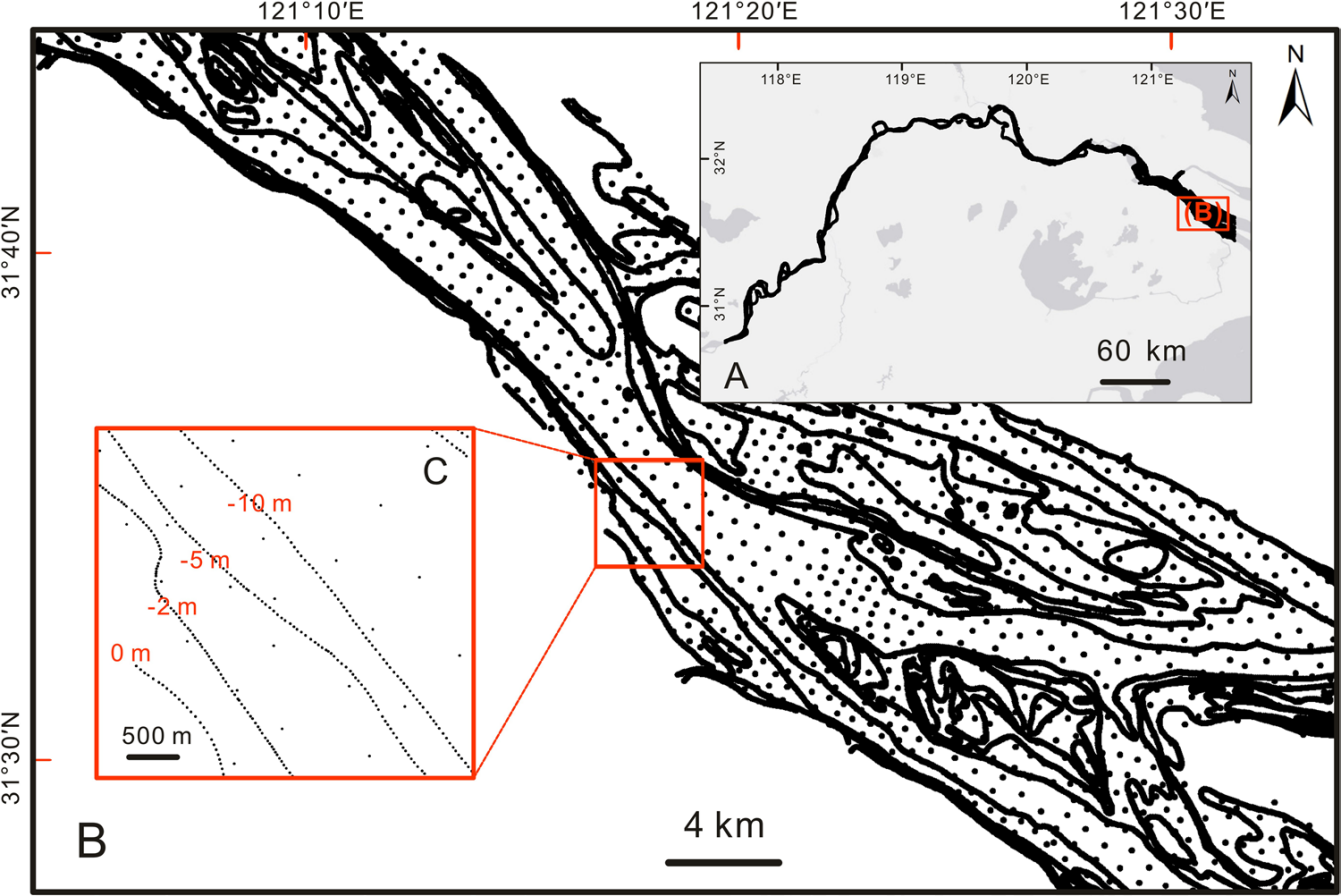
Digitized water depth points and isobaths from the 1998 navigation charts. The water depth data cover bank to bank along the 565-km channel from Datong to Wusongkou. The digitized navigation charts have an average data density of 90–100 depth points per square kilometer.

We collected 52 navigational charts of 1998 and 2013 surveyed by the Chang Jiang Waterway Bureau (CJWB) from Datong to Wusongkou. Each chart covered approximately 11 km of the river channel. These navigation charts included water depth points with 500 m distance between two adjacent points (Fig. 2 and Supplementary Fig. S1). The water depth data for 1998 were generated from the charts of 1994, 1995 and 1998, and the scales of these maps were 1:40,000 and 1:60,000 (Supplementary Tables S1 and S2) which collected according to Specifications for Port and Waterway Engineering Survey published in 1994 (JTJ203-94). The water depth data for 2013 were derived from the charts of 2012 and 2013, and the scales of these maps were 1:20,000 to 1:40,000 according to Specifications for Port and Waterway Engineering Survey published in 2012 (JTS131-2012). All bathymetry data were carried out with a vertical error of about ± 0.2 m when water depth (H) was ≤20 m and of ± 0.01 H when water depth was >20 m.

The reference datum used in the 1998 and 2013 charts are same. For example, the reference datum upstream of Jiangyin (RK 160–565) is the navigational datum of the Lower Yangtze River; The reference datum downstream of Jiangyin (RK 0–160) is the theoretical lowest tide level. Sediment load and river discharge data at Datong Station from 1998 to 2015 were collected from the Changjiang Water Resources Commission of the Ministry of Water Resources (http://www.cjw.gov.cn). All the navigation chart maps were processed in ArcGIS 10.4 (ESRI, Redlands, CA).

### Estimation of channel width changes

Channel width along the Lowermost Yangtze River could have extensively changed during the past two decades due to extensive river engineering work (i.e., shoreline protection and coastal reclamation). Previous studies found that human modifications of a large alluvial river channel could lead to considerable adjustments of bed morphology^26^. Therefore, we first assessed the channel width from Datong to Wusongkou in 1998 and in 2013 using the DEMs data. A total of 167 cross sections were selected along the 565-km river reach (Fig. 1). Downstream RK 70, the channel width is the top width of the cross section when the water level is equal to the theoretical lowest tide level, while upstream RK 70, the channel width is the top width of the cross section when the water level is equal to the navigation datum (Supplementary Fig. S2).

### Assessment of bed deformation

With the same reference datum, changes of water depth are caused by riverbed elevation changes body^27,28^. Therefore, by comparing the changes in water depth between 1998 and 2013, we can estimate the sediment deposition and erosion on the bed. For instance, the decreased water depth indicates sediment deposition, while the increased water depth indicates eroded riverbed.

In this study, the navigational charts were georeferenced using 5–9 fixed landmarks with an error less than 0.01 cm in ArcGIS 10.4. For generating high-quality digital elevation models (DEMs), in addition to original water depth points, depth points were also extracted from the isobaths of 0 m, −2 m, −5 m, and −10 m with a spacing of 80–150 m between two points. As a result, each digitized navigational chart has an average density of 90–100 depth points per square kilometer. The water depth data were interpolated to DEMs in 1998 and 2013 with 25 m × 25 m spatial resolution using kriging interpolation technique. For a detailed interpretation of the riverbed elevation changes, the 565-km long DEM of the study reach was further divided into 57 sub-reaches, each presenting a 10-km long channel. The change of average bed elevation and average deposition/erosion depth were quantified for each subreach from RK0 to RK560. The last segment was a 5-km long subreach, i.e., from RK 560 to RK 565.

The error of the estimated results is mainly from navigational charts data and the interpolation, which was estimated to be less than 10%^29,30^.

### Multi-beam data

Riverbed bedforms along the main navigation channel from Wusongkou to Datong were examined using a SeaBat 7125 multi-beam echo sounder system (Teledyne Reson, Slangerup, Denmark) under a discharge of 47,000–30,000 m^3^ s^−1^ during July 27 and August 14, 2015, and 31,000–23,000 m^3^ s^−1^ during September 09 and 18, 2016. The SeaBat 7125 multi-beam echo sounder system contains a compass, motion sensor, positioning system, depth sensor, and sound velocity to control the data accuracy. The compass is used to get a heading. The motion sensor measures the attitude (roll, pitch, and heave) of the boat. An external access DGPS positioning system was used to collect the position information. The depth sensor is used in the sea level computation for two purposes. One is to obtain the sea level and the other is obtain the depth of the boat. The sound velocity sensors are used to obtain a sound velocity in time. The principle of multi-beam is a proper phase or time delay for signals received from different sources in the acoustic array which can guide the main lobe to a specific direction. This sounder had an operational working frequency of 200 kHz/400 kHz. Under the mode of 400 kHz, the central beam angle is 0.5° and the maximum ping rate is (50 ± 1) Hz. It has 512 beams at 400 kHz and 256 beams at 200 kHz. The theoretical depth resolution is 6 mm, and typical measurement depth is 0.5 m to 150 m at 400 kHz. Under the mode of 400 kHz, the along-track transmit beam-width is 1° and the across-track receive beam-width is 0.5°. During the surveying, a Trimble real-time differential global positioning system (Trimble Inc., Sunnyvale, CA) was used to control the position accuracy at the decimeter level. The traveling speed of measuring boat was controlled to be as constant as possible at 1–3.5 m s^−1^. The threshold of maximum ping rate was 20 Hz which can automatically adjust with water depth. The real Ping rate was not less 9 times per second. Thus, the precision of the successive measurements along the direction of the boat during 0.1–0.4 m. The swath width can cover more than 5–6 times of the water depth. The maximum water depth is about 50 m in the lower Yangtze along the main navigation channel. The 400 kHz work mode was chosen in the field, thus the swath width was no more than 300 m with 512 beams. As a result, the precision of the successive measurements perpendicular to the direction of the boat is 0.6 m. The final point cloud data were processed by sound speed correction. Meanwhile, the calibrations of the roll, pitch, and yaw were conducted. Abnormal beams were removed in the editing module using the PDS 2000 software (Version 3.9.1.7). Finally, the mapping of riverbed bedforms was directly built by the processed point cloud data, and the resolution was set to 1 m × 1 m.

## Results

### Changes in channel width and bed elevation

**Figure 3:**
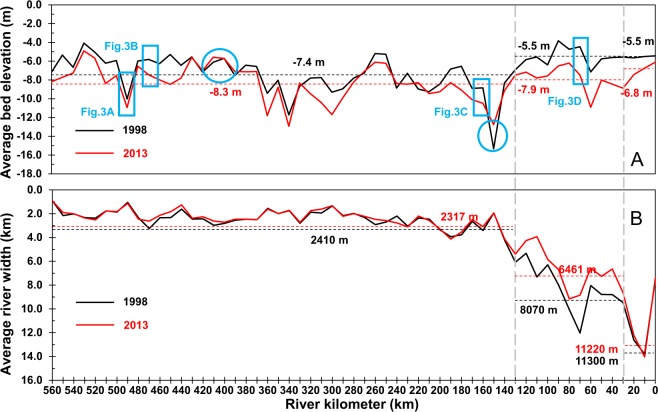
Average bed elevation (**A**) and average river width (**B**). (**A**) The average bed elevation in 1998 (black solid line) and 2013 (red solid line), which were calculated through dividing the estimated channel volume change for each subreach by the subreach surface area. (**B**) Average river width of the Lowermost Yangtze River in 1998 (black solid line) and 2013 (red solid line). The black and red dotted lines are the average river width in 1998 and 2013 from RK 565 to RK 130, RK 130 to RK 30, and RK 30 to RK 0, respectively. The reference datum was kept same for 1998 and 2013. The navigational datum of the Lowermost Yangtze River was used as a reference datum for the reach upstream of Jiangyin (RK 160–565) and the theoretical lowest tide level was used as a reference datum for the reach downstream of Jiangyin (RK 0–160). Black and red dotted lines are the average river width in 1998 and 2013 from RK 565 to RK 130, RK 130 to RK 30, and RK 30 to RK 0, respectively. The blue boxes show the locations of the multi-beam photos in Fig. 3. Blue circles indicate the reaches with bed aggradation.

Based on 167 cross-sectional measurements in 1998 and 2013, we found that on average, the 565-km Lowermost Yangtze River has narrowed by 370 m in the past 15 years. The change in channel width mainly occurred in the lower reach of the Lowermost Yangtze River. Specifically, the channel width of the river reach from RK 130 to RK 30 decreased by 1609 m (i.e., from 8070 m to 6461 m) (Fig. 3B), while the channel width between RK 565 and RK 130 only slightly reduced, i.e., from 2410 m in 1998 to 2317 m in 2013. The river reach below RK 30 showed little change in its channel width, i.e., from 11,300 m in 1998 to 11,220 m in 2013.

There was a significant reduction in bed elevation from 1998 to 2013 along the entire 565-km Lowermost Yangtze River (Fig. 3A). Overall, the average bed elevation decreased by 1.2 m from −7 m to −8.2 m during 1998–2013. The largest bed degradation occurred in the reach from RK 130 to RK 30, specifically, a 2.4 m depth degradation from −5.5 m in 1998 to −7.9 m in 2013. There was relatively much smaller bed degradation for the reach upstream of RK 130, i.e., a 0.9 m degradation from −7.4 m to −8.3 m for the same period. Bed aggradation mainly occurred in two short reaches, RK 145–155 and RK 395–425.

### Multi-beam riverbed measurements

**Figure 4:**
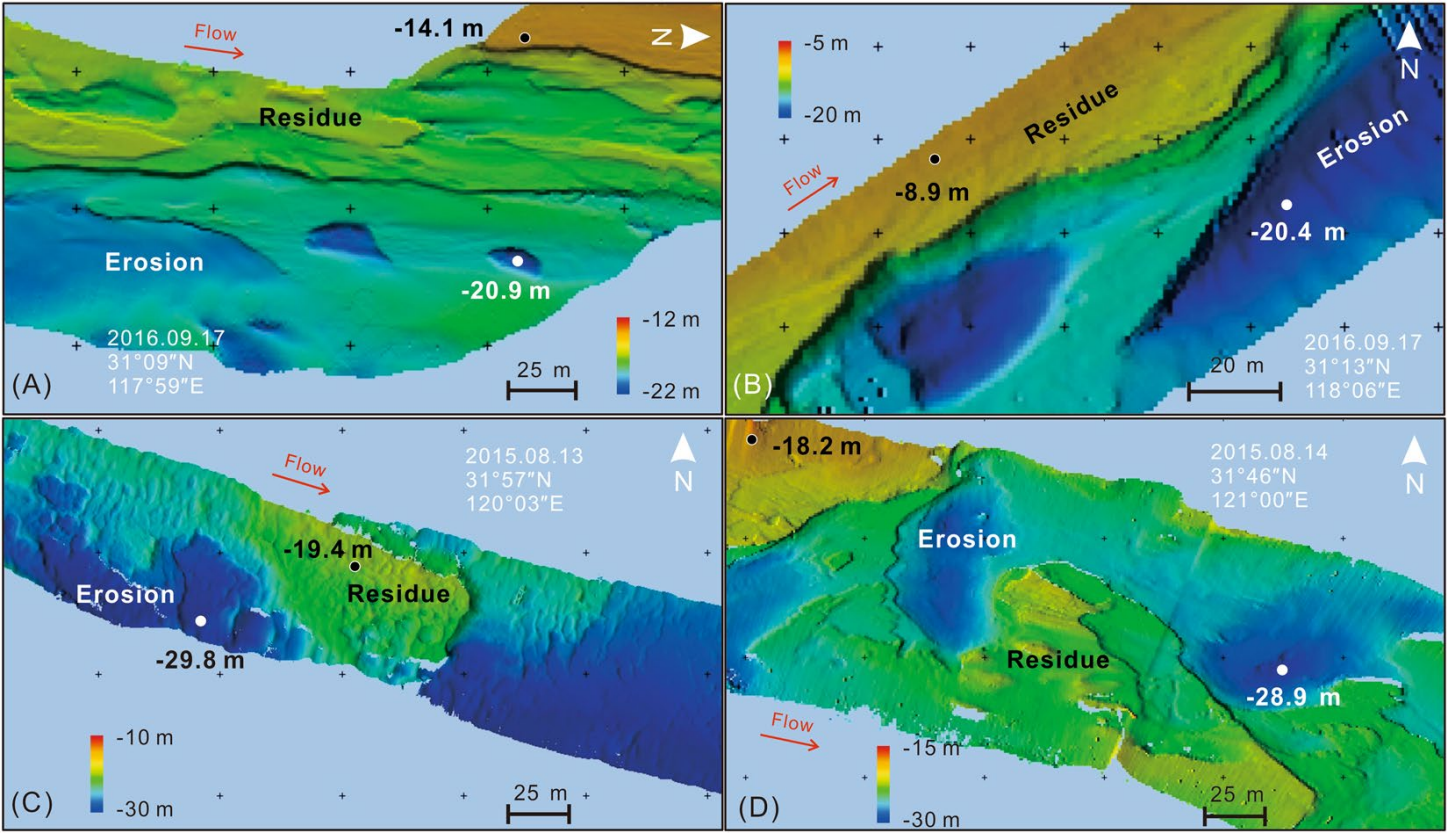
Multibeam measurements in 2015 and 2016 showing riverbed morphology of the main channel from Wusongkou to Datong of the Lowermost Yangtze River. (**A**) Layered erosion structure at the segment of RK 490. (**B**) Erosional flutes with the block residues at the segment of RK 470. (**C**) Erosional holes with the block residues in Jiangyin, dunes development with wavelength of 7–12 m, height of 0.4–0.7 m, and (**D**) Xuliujing, between RK 160 and RK 65. The values near white dots show the deepest bed elevation in erosional holes or flutes. The values near black dots show the bed elevation of residue (i.e., the current elevation). The maximum scour depth was calculated as the difference of bed elevation between erosional holes (flutes) and residue. All bed elevation values in this figure were calculated according to the water surface under a discharge of 31,000 m^3^ s^−1^ during August 13–14, 2015, and 25,000 m^3^ s^−1^ on September 17, 2016. Note: the maps were created using PDS 2000 3.9.1.7 (www.teledyne-pds.com) and CorelDRAW Graphics Suite 2017 (www.coreldraw.com).

The 681-km multi-beam measurements along the main navigation channel of the Lowermost Yangtze River were used to verify the current riverbed topography. Approximately 63.3% of the surveyed reach developed dunes, 21.8% showed eroded surface, and 14.9% was a flat riverbed^31^. Dunes distributed along the entire studied reach and the distribution density of very large dunes (wavelength greater than 100 m) showed a decreasing trend from Datong to the river mouth. Statistics data show that the flat riverbed mainly developed downstream of Xuliujing (RK 70–0). In this segment, the length of flat riverbed accounted for 72.2% of the total flat riverbed length in the 565-km Lowermost Yangtze River. More than 10-m deep riverbed erosion was observed at several segments (Fig. 4). For instance, an average erosion depth of 11.5 m was found at RK 470 (Fig. 4B), whereby the riverbed depth surrounding the erosional holes was 8.9 m, while the maximum depth of the erosional holes was 20.4 m with a discharge of 25,000 m^3^ s^−1^ recorded at the Datong station. Similar large erosional phenomena were also found in other river segments, e.g., RK 490, RK 160, and RK 65 (Fig. 4).

### Riverbed erosion and deposition estimates

**Figure 5:**
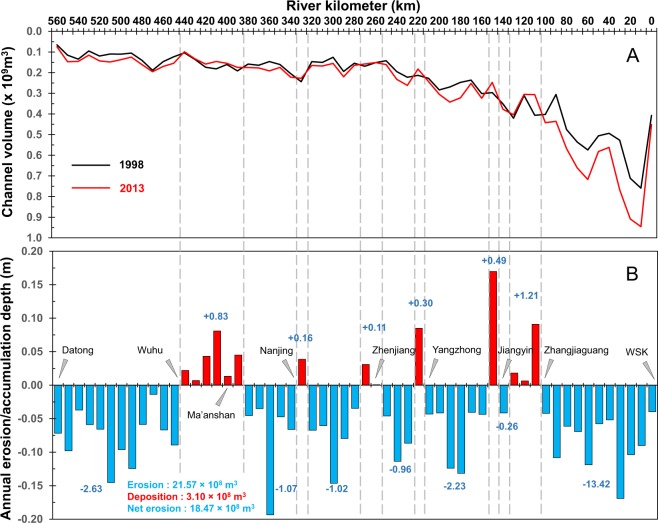
Average channel volume change (**A**) and average annual erosion and deposition rate (**B**). (**A**) Comparison of average river depth between 1998 (black line) and 2013 (red line) of the Lowermost Yangtze River. (**B**) Net erosion (blue bars) and deposition (red bars) histograms. Sediment deposition mainly occurred in two reaches (i.e. RK 100–150 and RK 380–440). Blue texts indicate volume change of the reaches.

The findings from this study show that the final 565 km of the Lowermost Yangtze River has undergone excess riverbed erosion in the past 15 years (Fig. 5), resulting in an average erosion of 0.05 m per year in the Lowermost Yangtze River (Fig. 5A). However, the erosion rates varied largely from 0.01 to 0.19 m per year along the river reach. The average bed degradation is 0.04 m per year in the upstream 465 km, while the value increases to 0.08 m per year in the final 100 km (Fig. 5B). Based on the changes in river width and bed elevation, we quantified a total erosion volume of 2.157 × 10^9^ m^3^. The deposition was found only in two reaches, i.e., RK 100–150 and RK 380–480, with a range of 0–0.17 m. The total deposition volume was much smaller (0.310 × 10^9^ m^3^) than that of erosion (Fig. 5B). Hence, a net change in volume of the riverbed was 1.847 × 10^9^ m^3^ from 1998 to 2013, i.e., 3.3 × 10^6^ m^3^/km. This enormous erosion would represent an equivalent of 2.59 billion metric tons of sediment, assuming a bulk density of 1.4 t/m^3^ for the riverbed material. It is worth noting that the erosion volume downstream of RK 100 accounted for 72.7% (or 1.342 × 10^9^ m^3^) of the entire net erosion volume (1.847 × 10^9^ m^3^) of the Lowermost Yangtze River.

## Discussion

### Impact factors of riverbed deformation

Riverbed deformation of an alluvial river is subject to governing conditions that include human activities and changes of sediment load, river discharge, and climate^32–34^. As a large alluvial river, the final 565 kilometers of the Yangtze River can be affected by many factors and the riverbed deformation mechanism is complicated^35–37^. The results from this study demonstrate that the Lowermost 565-km Yangtze River experienced excess riverbed erosion from 1998 to 2013. Before the construction of the Three Gorges Dam (TGD), channel bed erosion downstream of it was predicted extending to the river mouth (i.e., 1600-km long channel). However, a recent study found that the bed erosion downstream of the TGD would very likely cease at the point about 400 km downstream of the dam^21^. Much coarser bed sediment and significantly reduced bed slope are main contributors to the cessation of the further downstream erosion. A previous 1-D modeling analysis of bed profiles downstream of dams also revealed that the bed erosion owning to sudden sediment reduction propagates extremely slow (i.e., several centuries) to the downstream in a large alluvial river^38^. Located about 1000 km downstream of the TGD, the observed excess bed erosion in this study seems unlikely to be caused solely by the closure of the TGD in 2003.

The largest deepening of riverbed occurred between RK 130 and RK 30, where the channel also narrowed greatly. Narrowing channels leading to bed erosion has been observed in many alluvial river systems throughout the world^39–41^. In fact, the river channel width of the entire Lowermost Yangtze River reduced by 9.1% from 1998 to 2013 (Fig. 3B) because of channel bar growth, reclamation of shoreline, and construction of dikes and revetments (Supplementary Tables S3 and S4). Between RK 130 and RK 30, the channel width reduced by nearly 20% (or 1609 m). This should have significantly increased the flow velocity, resulting in the bed degradation in this river reach.

In terms of erosion volume, our result shows that the channel bed downstream of RK 100 was mostly eroded, accounting for 72.7% (or 1.342 × 10^9^ m^3^) of the entire net erosion (1.847 × 10^9^ m^3^) of the Lowermost Yangtze River. Except for the channel width reduction, the bed degradation could also be closely related to the backwater effects. In large alluvial rivers, the Lowermost reaches tend to be scoured during high flow due to the drowdown effects. This can be seen from the modeling studies^42,43^ and the recently observed bed erosion in the Lowermost Mississippi River^44^. In fact, the channel bed in the last 50 km of the Yangtze has experienced erosion since 1978, according to a long-term bathymetry study by Luan *et al*.^45^. The largest erosion was found between 1986 and 1997 when frequent floods occurred. Therefore, the flood effects on bed morphology in this reach could not be neglected. Particularly, historical floods occurred in 1998 and 1999 with the peak discharge over 80,000 m^3^/s. These floods may have also contributed to the bed erosion downstream of RK 100. In addition, this reach is influenced by both channel flow and tide current in the same direction during ebb tide^46^. This can aggravate the channel erosion. Finnally, although it has been reported that the average sea level rise rate is 2.9 mm/yr along the Chinese coasts btween 1950 and 2013 (SOA, 2013). It is not clear how much the bed erosion near the river mouth was affected by the sea level rise. The slightly degrading in the reach between RK 565 and RK 130 is consistent with the bed erosion upstream between Hankou and Datong^47^. In-channel sand mining can be a possible reason for this trend^48^.

The 1.847 × 10^9^ m^3^ eroded sediment in the Lowermost Yangtze River may have deposited in the river mouth and submerged delta front. Dai and others (2014) found that the Yangtze River submerged delta experienced high sediment accumulation during 2002–2009, although large reduction of suspended sediment occurred after the closure of Three Gorges Dam in 2003 (i.e., from 4.33 × 10^8^ t during 1950–2000 to 1.39 × 10^8^ t during 2003–2015, Supplementary Fig. S3). The large eroded amount of sediment in the Lowermost Yangtze River should have been an important source for the accumulation. It is also plausible that sediments eroded from the riverbed are coarser and heavier than suspended sediment, making them more likely to deposit near the river mouth. This was recently observed by Yang *et al*.^49^, whereby they found that the average median size of the sediment in the river mouth increased from 8.0 µm in 1982 to 15.4 µm in 2012.

While average channel width of the reach RK 130–100 reduced by 20% (or 1,609 m) from 1998 to 2013 (Fig. 3B), a deposition of 0.121 × 10^9^ m^3^ occurred in the 30-km reach (Fig. 5B). Several engineering projects in the past 15 years (Supplementary S4) may have contributed to the deposition. For instance, a sandbar consolidation project at Rugaoqunsha (Supplementary S3) caused kilometers of sedimentation along the left bank of the 30-km reach. As a result, the right bank occurred excess riverbed erosion up to 3 m (Fig. 3A). A recent study on foundation scours of the large bridges in the studied river reach pointed out that the riverbed erosion phenomenon after the TGD closure has been stronger^31^. Although we are unable to do any comparison on grain size before and after the TGD construction, a study by Luo *et al*.^11^ has reported such a change and has suggested that the impact of the Three Gorges Dam on grain size in the middle and lower Yangtze River would continue after 2011. It is therefore likely that excess bed erosion in this river reach would continue in the future.

### Channel evolution mechanism response to natural and human activities

The sedimentation mechanism of upstream erosion and downstream-ward aggradations is weakened in the Lowermost Yangtze River in the past 15 years. This reach is influenced by both channel flow and tide current in the same direction during^46^. However, the erosion and deposition pattern found in our study shows that the volume of erosion in upstream reach is 1.5–9.3 times than the volume of deposition in its downstream (Table 1). This phenomenon suggests that scoured sediment from the riverbed became the source of suspended sediment in the LmYR. Hence, a large amount of sediment was eroded from upstream and just part of them deposited in the downstream river channel. As a result, the sedimentation mechanism of upstream erosion and downstream-ward aggradations in the LmYR is weakened owing to the sediment decline in recent years.

**Table 1:** The erosion and deposition volume along the Lowermost Yangtze River.

Reach	RK 565–390	RK 390–330	RK 330–260	RK 260–220	RK 220–110	RK 110–0
Erosion volume (x 10^9^ m^3^)	0.263	0.107	0.102	0.096	0.249	1.342
Deposition volume (x 10^9^ m^3^)	0.083	0.016	0.011	0.030	0.17	—
Erosion/Deposition	3.2	6.7	9.3	3.2	1.5	—

**Figure 6:**
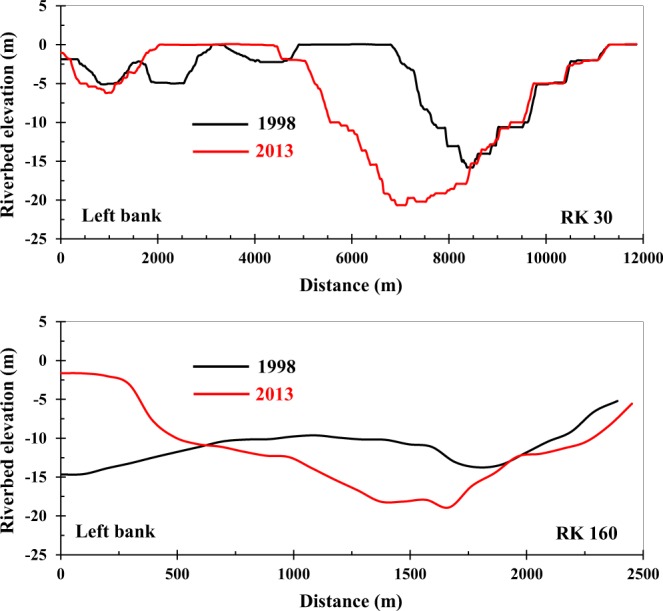
Two channel cross sections of the Yangtze River at RK30 and RK160. Red lines are cross sections in 2013 and black lines are cross sections in 1998.

**Figure 7:**
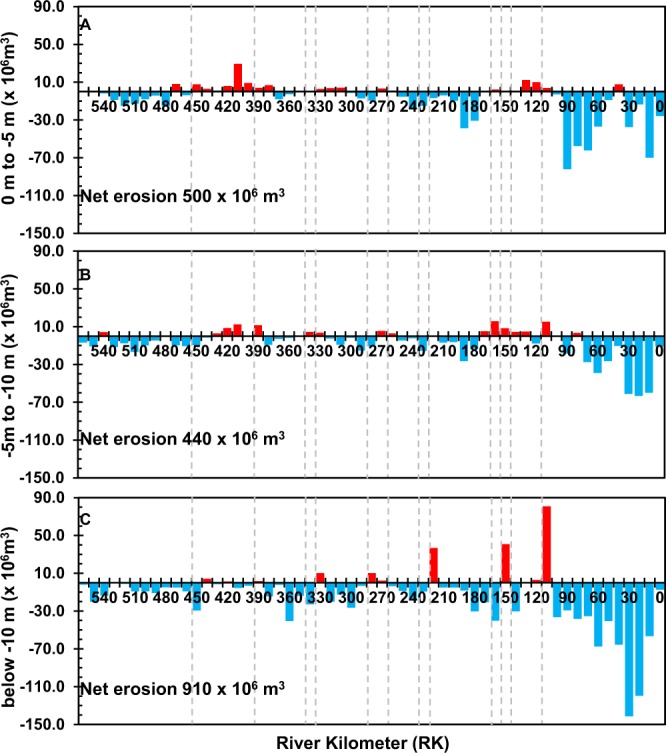
Erosion, deposition, and net change (i.e. erosion – deposition) of channel volume along the final 565 kilometers of the Yangtze River. Blue and red histograms indicate erosion and deposition volumes, respectively, between 0 m to −5 m (**A**), between −5 m to −10 m (**B**), and below −10 m (**C**) in the Lowermost Yangtze River during 1998–2013.

A detailed analysis of the erosion and deposition process in different water levels shows that the river reaches of deeper than 10 m (Fig. 6) in the last 100 km were the main erosion areas in the Lowermost Yangtze River in the past 15 years, accounting for 49% of the total erosion volume (Table 2). Detailed erosion and deposition volume data of 0 – −5 m, −5 – −10 m, and below −10 m also showed similar results (Fig. 7). This suggests that reaches deeper than 10 m were the main erosion areas of the riverbed.

**Table 2:** The river channel surface area and volume changes of the Lowermost Yangtze River during 1998–2013.

Year	Area			Volume		
	0 to −5 m	−5 to −10 m	below −10 m	0 to −5 m	−5 to −10 m	below −10 m
	(x 10^9^ m^2^)	(x 10^9^ m^2^)	(x 10^9^ m^2^)	(x 10^9^ m^3^)	(x 10^9^ m^3^)	(x 10^9^ m^3^)
1998	1.02	0.52	0.73	7.82	4.87	1.7
2013	0.72	0.57	0.78	8.35	5.31	2.61
1998–2013	0.3	−0.05	−0.05	−0.5	−0.44	−0.91

Note: Positive values indicate riverbed deposition while negative values indicate riverbed erosion.

Contributors to the riverbed erosion in the last 565-km Low most Yangtze River can include channel width reduction, backwater effects, tide and wave, and in-channel sand mining activities. In addition, due to the construction of TGD, a large amount of sediment was trapped in the upstream of the YR after 2003, which could contribute to increasing sediment transport capacity in the downstream river reach^45,50,51^. It is not clear, however, how long it will take until a new hydromorphological equilibrium is reached in the Lowermost Yangtze River under such a complex of human and natural impacts. Modeling analysis in the future could probably help answer the questions of how channel width change can affect bed morphology and at what point bed grain size distribution will become stable enough to withstand further scouring tendencies in this large alluvial river.

## Conclusions

This study assessed river width and bathymetric changes in the last 565 kilometers of the Lowermost Yangtze River between 1998 and 2013. The assessment is the first comprehensive analysis of channel deformation of this large alluvial river following the Three Gorges Dam closure in 2003, after which riverine sediment loading declined significantly. We found that on average, the river reach reduced by approximately 370 m (or 9.1%) in width and scoured about 1.2 m in bed elevation from 1998 to 2013. The total net erosion volume from the 565-km riverbed amounted to 1.85 billion m^3^, an equivalent of 2.59 billion metric tons of sediment assuming a bulk density of 1.4 t/m^3^ for the riverbed material in the past 15 years. Of the entire studied river reach, 425 kilometers have undergone substantial erosion (2.16 billion m^3^, annual average erosion rate ranging between 0.01 and 0.19 m) while the other 140 kilometers have experienced marginal deposition (0.31 billion m^3^, annual average deposition rate ranging between 0 and 0.17 m). We attribute this excess erosion to the rapid reduction in sediment load following the closure of the Three Gorges Dam in 2003. The average bed elevation and deepest trough curves changed following the erosion and deposition patterns. Severe channel erosion up to 10 m deep was observed at many locations, especially in the last downstream 100 kilometers, showing the seriousness of channel erosion of the Lowermost Yangtze River in the recent 15 years. The findings strongly indicate that the channel erosion will continue progressing, potentially threatening the long-term stability of this large alluvial river and its deltaic and continental shelf development.

## Electronic supplementary material

Supplementary Information
