# Supplementary material for: Riverbed erosion of the final 565 kilometers of the Yangtze River (Changjiang) following construction of the Three Gorges Dam
Source: Sci Rep. 2018 Aug 9;8:11917. doi: 10.1038/s41598-018-30441-6 (PMC6085297; doi:10.1038/s41598-018-30441-6)
Supplement: Supplementary file 1 — Supplementary Information [file 41598_2018_30441_MOESM1_ESM.doc]

Supplementary Information for:

# **Riverbed erosion of the last 565 kilometers of the Yangtze River (Changjiang) following construction of the Three Gorges Dam**

Shuwei Zheng^1, 2^, Yijun Xu^2,*^, Heqin Cheng^1,*^, Bo Wang^2^, Wei Xu^1^, Shuaihu Wu^1^

^1^State Key Lab of Estuarine & Coastal Research, East China Normal University, Shanghai 200062, China

^2^School of Renewable Natural Resources, Louisiana State University Agricultural Center, 227 Highland Road, Baton Rouge, LA 70803, USA

*corresponding authors: Y. Jun Xu. Professor, email: [yjxu@lsu.edu](mailto:yjxu@lsu.edu);

Heqin Cheng. Professor, email: [hqch@sklec.ecnu.edu.cn](mailto:hqch@sklec.ecnu.edu.cn)


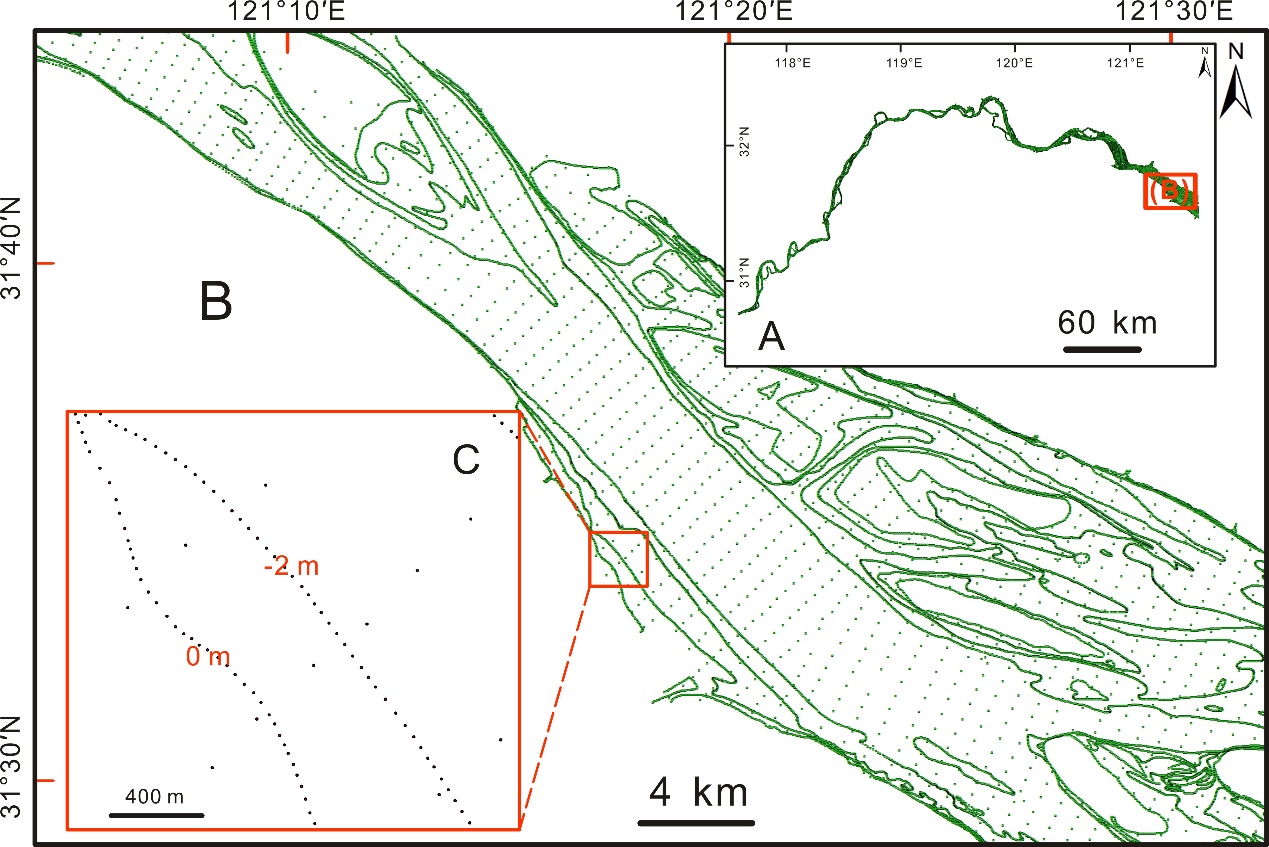


**Figure S1. Digitized water depth points and isobaths from the 2013 navigation charts from Datong to Wusongkou.** The total number of water depth points is 200,361 along the 565-km river channel.

**Figure S2. Cross sections of river channel at RK70, RK150, RK425, and RK550.** Red lines are cross sections in 2013 and black lines are cross sections in 1998. In this study, channel width is calculated by the DEM built by the navigational charts datasets. The channel width values in the reach downstream of RK 70 are equal to the cross section length of the theoretical lowest tide level, and the channel width values in the reach upstream of RK 70 are equal to the cross section length of the navigation datum. This means, the reference water surface of 0 m is the theoretical lowest tide level downstream RK 70 and Navigation datum upstream RK 70, respectively.

**Figure S3.** Changes in flow (A), sediment load (B), sediment concentration (C), and median grain size (D) at Datong (2000–2015) of the Yangtze River. While annual river flow showed insignificant change in the past 15 years (2000–2015), both sediment concentration and median grain size declined largely following the closure of the Three Gorges Dam (TGD) in 2003. Although the grain size quickly recovered from 8 μm in 2004 to an average value of 10.5 μm during 2006-2015, it is smaller than the average value of 17 μm during 1976-2000. As a result, annual sediment discharge to the lower river reach reduced largely.

**Table S1. Information on navigational charts of the Lowermost Yangtze River published in 1998**.

| Published | Map Title | number | Scale | Datum | corrdinate system | RK |
| --- | --- | --- | --- | --- | --- | --- |
| 1998 | Wusongkou to Shidongkou | 9 | 1:60,000 | lowest normal low water | 1954, Beijing | 0-15 |
| 1998 | Shikongkou to Qiyakou | 10 | 1:60,000 | lowest normal low water | 1954, Beijing | 15-40 |
| 1998 | Baimaosha and nearby waterway | 11 | 1:60,000 | lowest normal low water | 1954, Beijing | 40-55 |
| 1998 | Qianjingkou to Xizhou | 12 | 1:60,000 | lowest normal low water | 1954, Beijing | 55-75 |
| 1998 | Xizhou to Langshan | 13 | 1:60,000 | lowest normal low water | 1954, Beijing | 75-95 |
| 1998 | Nantong and nearby area | 14 | 1:60,000 | lowest normal low water | 1954, Beijing | 95-115 |
| 1998 | Xingzheng to Liuwei | 15 | 1:40,000 | lowest normal low water | 1954, Beijing | 115-135 |
| 1998 | Zhangjiagang and nearby area | 16 | 1:40,000 | lowest normal low water | 1954, Beijing | 135-150 |
| 1998 | Dahe port to Rugaowei | 17 | 1:40,000 | navigation datum | 1954, Beijing | 150-165 |
| 1998 | Rugaowei to Lu'anzhou | 18 | 1:40,000 | navigation datum | 1954, Beijing | 165-185 |
| 1998 | Lu'anzhou to Guochuan port | 19 | 1:40,000 | navigation datum | 1954, Beijing | 185-205 |
| 1998 | Guochuan port to Tiepi port | 20 | 1:40,000 | navigation datum | 1954, Beijing | 205-225 |
| 1998 | Tiepi port to Hechuangzhou | 21 | 1:40,000 | navigation datum | 1954, Beijing | 225-250 |
| 1998 | Hechuangzhou to zhengrunzhou | 22 | 1:40,000 | navigation datum | 1954, Beijing | 250-270 |
| 1998 | Shiyezhou and nearby area | 23 | 1:40,000 | navigation datum | 1954, Beijing | 270-290 |
| 1998 | Shiyezhou to Longtan | 24 | 1:40,000 | navigation datum | 1954, Beijing | 300-310 |
| 1998 | Longtan to Baguazhou | 25 | 1:40,000 | navigation datum | 1954, Beijing | 320-330 |
| 1998 | Baguazhou and nearby waterway | 26 | 1:40,000 | navigation datum | 1954, Beijing | 330-340 |
| 1998 | Jiangxinzhou and nearby area | 27 | 1:40,000 | navigation datum | 1954, Beijing | 340-360 |
| 1998 | Jiangxinzhou to Xinjizhou | 28 | 1:40,000 | navigation datum | 1954, Beijing | 360-380 |
| 1998 | Xinjizhou to Xinhekou | 29 | 1:40,000 | navigation datum | 1954, Beijing | 380-400 |
| 1998 | Xinhekou to Hongzhuang | 30 | 1:40,000 | navigation datum | 1954, Beijing | 400-420 |
| 1998 | Hongzhuang to Wuhu | 31 | 1:40,000 | navigation datum | 1954, Beijing | 420-440 |
| 1998 | Wuhu to Baodingwei | 32 | 1:40,000 | navigation datum | 1954, Beijing | 440-465 |
| 1998 | Baodingwei to Tianranzhou | 33 | 1:40,000 | navigation datum | 1954, Beijing | 465-480 |
| 1998 | Tianranzhou to Jinniudu | 34 | 1:40,000 | navigation datum | 1954, Beijing | 480-505 |
| 1998 | Jinniudu to Chengdezhou | 35 | 1:40,000 | navigation datum | 1954, Beijing | 500-530 |
| 1998 | Chengdezhou and nearby area | 36 | 1:40,000 | navigation datum | 1954, Beijing | 530-545 |
| 1998 | Henggang to Chongwenzhou | 37 | 1:40,000 | navigation datum | 1954, Beijing | 540-570 |

**Table S2. Information on navigational charts of the Lowermost Yangtze River published in 2013**.

| Published | Map Title | number | Scale | Datum | corrdinate system | RK |
| --- | --- | --- | --- | --- | --- | --- |
| 2013 | Wusongkou to Liuhekou | 1 | 1:50,000 | lowest normal low water | 1954, Beijing | 0-20 |
| 2013 | Liuhekou to Liuwenjing | 2 | 1:40,000 | lowest normal low water | 1954, Beijing | 30-40 |
| 2013 | Liuwenjing to Baimaohe | 3 | 1:40,000 | lowest normal low water | 1954, Beijing | 40-60 |
| 2013 | Baimaohe to Laohong port | 4 | 1:40,000 | lowest normal low water | 1954, Beijing | 60-80 |
| 2013 | Laohong port to Tiansheng port | 5 | 1:40,000 | lowest normal low water | 1954, Beijing | 80-100 |
| 2013 | Tiansheng port to Duanshan | 6 | 1:40,000 | lowest normal low water | 1954, Beijing | 110-120 |
| 2013 | Duanshen to Erbizui | 7 | 1:40,000 | lowest normal low water | 1954, Beijing | 130-150 |
| 2013 | Erbizui to Lianchengzhou | 8 | 1:40,000 | navigation datum | 1954, Beijing | 160-180 |
| 2013 | Lianchengzhou to Shisiwei | 9 | 1:40,000 | navigation datum | 1954, Beijing | 180-200 |
| 2013 | Shisiwei to Luochengzhou | 10 | 1:40,000 | navigation datum | 1954, Beijing | 200-230 |
| 2013 | Luochengzhou to Jiaoshan | 11 | 1:40,000 | navigation datum | 1954, Beijing | 230-260 |
| 2013 | Jiaoshan to shierwei | 12 | 1:40,000 | navigation datum | 1954, Beijing | 270-290 |
| 2013 | Shierwei to Qixiashan | 13 | 1:40,000 | navigation datum | 1954, Beijing | 300-320 |
| 2013 | Qixiashan to Zhongshan pier | 14 | 1:40,000 | navigation datum | 1954, Beijing | 320-350 |
| 2013 | Nanjing port area | 14-1 | 1:20,000 | navigation datum | 1954, Beijing | 340-350 |
| 2013 | Zhongshan pier to Qiudingzhou | 15 | 1:40,000 | navigation datum | 1954, Beijing | 350-370 |
| 2013 | Qiudingzhou to Hejiazhou | 16 | 1:40,000 | navigation datum | 1954, Beijing | 380-400 |
| 2013 | Hejiazhou to Chenjiazhou | 17 | 1:40,000 | navigation datum | 1954, Beijing | 400-430 |
| 2013 | Chenjiazhou to Dahewei | 18 | 1:40,000 | navigation datum | 1954, Beijing | 430-460 |
| 2013 | Dahewei to Wanjiatan | 19 | 1:40,000 | navigation datum | 1954, Beijing | 460-480 |
| 2013 | Wanjiangtan to Zhangjiazhou | 20 | 1:40,000 | navigation datum | 1954, Beijing | 490-520 |
| 2013 | Zhangjiazhou to Hejiachang | 21 | 1:40,000 | navigation datum | 1954, Beijing | 530-550 |
| 2013 | Hejiachang to Nizhou | 22 | 1:40,000 | navigation datum | 1954, Beijing | 550-570 |

**Table S3. Information on major sandbars from the 2013 multi-beam channel survey**.

| Number | Name | RK | Type | Cover | Project |
| --- | --- | --- | --- | --- | --- |
| 1 | Ruifengsha | 0 | Mid-channel Bar | None | None |
| 2 | Xinliuhesha | 10 | Mid-channel Bar | None | Berm |
| 3 | Xiabiandansha | 20 | Mid-channel Bar | None | None |
| 4 | Xibiandansha | 30 | Mid-channel Bar | None | None |
| 5 | Baimaosha | 50 | Mid-channel Bar | Middle | Berm and Spur Dikes |
| 6 | Langshansha | 75 | Mid-channel Bar | Vegetation | Berm and Spur Dikes |
| 7 | Tongzhousha | 95 | Side Bar | Vegetation | Berm |
| 8 | Rugaoqunsha | 115 | Multiple Bars | Town and Farmland | Berm |
| 9 | Minzhu-Changsha | 125 | Mid-channel Bar | Farmland | Berm |
| 10 | Fujiangsha | 140 | Attached Bar | Farmland | Berm |
| 11 | Lu'Anzhou | 185 | Side Bar | Farmland | Berm |
| 12 | Tianxingsha | 190 | Side Bar | Vegetation | Berm |
| 13 | Luochengzhou | 230 | Mid-channel Bar | Framland | Berm |
| 14 | Taipingzhou | 190-240 | Attached Bar | City and Farmland | Berm |
| 15 | Hechuangzhou | 250 | Mid-channel Bar | Town and Farmland | Berm |
| 16 | Jiaobeizhou | 260 | Attached Bar | Farmland | Berm |
| 17 | Dingyizhou | 270 | Attached Bar | Farmland | Berm |
| 18 | Shiyezhou | 280-290 | Mid-channel Bar | Town and Farmland | Berm |
| 19 | Baguazhou | 330-340 | Concave Bar | Town and Farmland | Berm |
| 20 | Qianzhou | 350 | Side Bar | Vegetation | Berm |
| 21 | Meizizhou | 350-360 | Side Bar | Town and Farmland | Berm |
| 22 | Xinji-Xinshengzhou | 370-390 | Multiple Bars | Vegetation and Farmland | Berm |
| 23 | Xiaohuangzhou | 400 | Mid-channel Bar | Farmland and Vegetation | Berm |
| 24 | Jiangxinzhou | 400-420 | Multiple Bars | Town and Farmland | Berm |
| 25 | Chenjiazhou | 430 | Side Bar | Town and Farmland | Berm |
| 26 | Caoguzhou | 433 | Mid-channel Bar | Vegetation | Berm |
| 27 | Heisahzhou | 480 | Concave Bar | Town, Farmland and Vegetation | Berm |
| 28 | Tonglingsha | 510 | Attached Bar | Farmland and Vegetation | Berm |
| 29 | Zhangjiazhou | 520 | Attached Bar | Farmland | Berm |
| 30 | Chengdezhou | 530-540 | Mid-channel Bar | Town and Farmland | Berm |
| 31 | Tieban-Heyuezhou | 555 | Side Bar | Town and Farmland | Berm |
| 32 | Shazhou | 557 | Side Bar | Town and Farmland | Berm |

**Table S4. Large engineering projects conducted in the Lowermost Yangtze River from Datong to Wusongkou in the past 10 years**.

| Name | Built time | RK |
| --- | --- | --- |
| The Xinliuhe shoal protection project and Nanshatou channel submerged dike | 2007-2009 | 0 |
|
|
| Regulation scheme of Baimaosha | 2012-2014 | 50 |
| Regulation scheme of Tongzhousha | 2012-2013 | 80-100 |
| Regulation scheme of shuangjiansha | 2010-2012 | 130 |
| Manyusha shoal head protection engineering | 2010-2012 | 210 |
| Luocheng shoal protection engineering | 2011-2013 | 235 |
| The first phase regulation project of Jiangxinzhou-Wujiang waterway | 2009-2011 | 390-420 |
|
|
| Regulation scheme of Heishazhou waterway | 2007-2009 | 480 |
| The first phase regulation project of Tuqiao waterway | 2009-2012 | 540 |
